# Supplementary material for: Modeling glioblastoma heterogeneity as a dynamic network of cell states
Source: Mol Syst Biol. 2021 Sep 16;17(9):e10105. doi: 10.15252/msb.202010105 (PMC8444284; doi:10.15252/msb.202010105)
Supplement: Supplementary file 5 — Source Data for Figure 3 [file MSB-17-e10105-s001.zip › Figure3A_sourcedata/GSEA_3065/nosigdb_state1.GseaPreranked.1622718409535/FELDMAN_2020_LATEG1.html]

Details for gene set FELDMAN\_2020\_LATEG1[GSEA]

|  || Dataset | state1 |
| Phenotype | NoPhenotypeAvailable |
| Upregulated in class | na\_pos |
| GeneSet | FELDMAN\_2020\_LATEG1 |
| Enrichment Score (ES) | 0.5174972 |
| Normalized Enrichment Score (NES) | 1.8918978 |
| Nominal p-value | 0.0 |
| FDR q-value | 8.9732127E-4 |
| FWER p-Value | 0.016 |
Table: GSEA Results Summary

  

Fig 1: Enrichment plot: FELDMAN\_2020\_LATEG1      
 Profile of the Running ES Score & Positions of GeneSet Members on the Rank Ordered List

  

| PROBE | GENE SYMBOL | GENE\_TITLE | RANK IN GENE LIST | RANK METRIC SCORE | RUNNING ES | CORE ENRICHMENT || 1 | UBE2S |  |  | 19 | 0.634 | 0.0312 | Yes |
| 2 | CCND1 |  |  | 29 | 0.566 | 0.0599 | Yes |
| 3 | ANXA1 |  |  | 30 | 0.564 | 0.0895 | Yes |
| 4 | TMEFF2 |  |  | 36 | 0.526 | 0.1165 | Yes |
| 5 | FBXO32 |  |  | 39 | 0.523 | 0.1436 | Yes |
| 6 | PTTG1 |  |  | 40 | 0.520 | 0.1708 | Yes |
| 7 | CTSV |  |  | 45 | 0.491 | 0.1961 | Yes |
| 8 | STK17A |  |  | 47 | 0.483 | 0.2213 | Yes |
| 9 | HOXB2 |  |  | 57 | 0.455 | 0.2442 | Yes |
| 10 | PHLDA1 |  |  | 83 | 0.389 | 0.2620 | Yes |
| 11 | ARL4C |  |  | 85 | 0.386 | 0.2821 | Yes |
| 12 | TNFRSF12A |  |  | 104 | 0.364 | 0.2993 | Yes |
| 13 | ARHGAP29 |  |  | 120 | 0.346 | 0.3159 | Yes |
| 14 | UBE2C |  |  | 125 | 0.340 | 0.3333 | Yes |
| 15 | TPM1 |  |  | 132 | 0.335 | 0.3502 | Yes |
| 16 | FHL2 |  |  | 141 | 0.328 | 0.3665 | Yes |
| 17 | TAGLN |  |  | 147 | 0.326 | 0.3831 | Yes |
| 18 | CKS2 |  |  | 195 | 0.297 | 0.3938 | Yes |
| 19 | BIRC5 |  |  | 202 | 0.295 | 0.4086 | Yes |
| 20 | CKS1B |  |  | 346 | 0.242 | 0.4067 | Yes |
| 21 | ACAT2 |  |  | 385 | 0.229 | 0.4148 | Yes |
| 22 | RAB13 |  |  | 417 | 0.223 | 0.4233 | Yes |
| 23 | LBH |  |  | 435 | 0.220 | 0.4331 | Yes |
| 24 | CDC20 |  |  | 469 | 0.212 | 0.4408 | Yes |
| 25 | MYL12A |  |  | 495 | 0.207 | 0.4491 | Yes |
| 26 | TPX2 |  |  | 556 | 0.194 | 0.4531 | Yes |
| 27 | CCNB1 |  |  | 583 | 0.189 | 0.4603 | Yes |
| 28 | FDFT1 |  |  | 635 | 0.181 | 0.4646 | Yes |
| 29 | CITED2 |  |  | 683 | 0.175 | 0.4689 | Yes |
| 30 | UGCG |  |  | 691 | 0.173 | 0.4773 | Yes |
| 31 | PLK2 |  |  | 725 | 0.168 | 0.4827 | Yes |
| 32 | CNN3 |  |  | 727 | 0.168 | 0.4914 | Yes |
| 33 | EBP |  |  | 829 | 0.154 | 0.4891 | Yes |
| 34 | TSC22D1 |  |  | 881 | 0.147 | 0.4916 | Yes |
| 35 | HMGB3 |  |  | 907 | 0.144 | 0.4965 | Yes |
| 36 | CALD1 |  |  | 968 | 0.137 | 0.4976 | Yes |
| 37 | CDCA3 |  |  | 994 | 0.134 | 0.5021 | Yes |
| 38 | FLG |  |  | 1020 | 0.132 | 0.5064 | Yes |
| 39 | HOXB4 |  |  | 1141 | 0.119 | 0.5004 | Yes |
| 40 | CCNB2 |  |  | 1232 | 0.110 | 0.4969 | Yes |
| 41 | NEXN |  |  | 1233 | 0.110 | 0.5026 | Yes |
| 42 | SERPINE1 |  |  | 1250 | 0.108 | 0.5066 | Yes |
| 43 | LAYN |  |  | 1285 | 0.105 | 0.5087 | Yes |
| 44 | PRSS23 |  |  | 1356 | 0.099 | 0.5067 | Yes |
| 45 | MAD2L1 |  |  | 1394 | 0.095 | 0.5079 | Yes |
| 46 | CTGF |  |  | 1416 | 0.094 | 0.5107 | Yes |
| 47 | SORBS2 |  |  | 1525 | 0.087 | 0.5042 | Yes |
| 48 | EZR |  |  | 1549 | 0.086 | 0.5064 | Yes |
| 49 | HMGB2 |  |  | 1570 | 0.085 | 0.5088 | Yes |
| 50 | MEST |  |  | 1582 | 0.084 | 0.5120 | Yes |
| 51 | PBK |  |  | 1651 | 0.079 | 0.5092 | Yes |
| 52 | LIMA1 |  |  | 1678 | 0.077 | 0.5106 | Yes |
| 53 | SOCS6 |  |  | 1687 | 0.076 | 0.5137 | Yes |
| 54 | NUSAP1 |  |  | 1736 | 0.073 | 0.5127 | Yes |
| 55 | CENPA |  |  | 1788 | 0.070 | 0.5111 | Yes |
| 56 | PRC1 |  |  | 1794 | 0.070 | 0.5143 | Yes |
| 57 | PLAU |  |  | 1801 | 0.069 | 0.5173 | Yes |
| 58 | CCNA2 |  |  | 1835 | 0.068 | 0.5175 | Yes |
| 59 | DLGAP5 |  |  | 2028 | 0.058 | 0.5009 | No |
| 60 | BEX1 |  |  | 2118 | 0.054 | 0.4947 | No |
| 61 | CYR61 |  |  | 2172 | 0.052 | 0.4920 | No |
| 62 | MVD |  |  | 2220 | 0.050 | 0.4898 | No |
| 63 | GADD45B |  |  | 2252 | 0.049 | 0.4892 | No |
| 64 | SMC4 |  |  | 2308 | 0.047 | 0.4860 | No |
| 65 | HERPUD1 |  |  | 2598 | 0.036 | 0.4584 | No |
| 66 | PAWR |  |  | 2666 | 0.034 | 0.4533 | No |
| 67 | ARID5B |  |  | 2743 | 0.032 | 0.4472 | No |
| 68 | MKI67 |  |  | 2760 | 0.031 | 0.4472 | No |
| 69 | CKAP2 |  |  | 2763 | 0.031 | 0.4487 | No |
| 70 | PRKAG2 |  |  | 3201 | 0.020 | 0.4051 | No |
| 71 | CDK1 |  |  | 3396 | 0.016 | 0.3861 | No |
| 72 | MYC |  |  | 3964 | 0.005 | 0.3284 | No |
| 73 | KLF6 |  |  | 4117 | 0.002 | 0.3130 | No |
| 74 | SRSF7 |  |  | 4413 | -0.003 | 0.2830 | No |
| 75 | FDPS |  |  | 4450 | -0.004 | 0.2795 | No |
| 76 | IDI1 |  |  | 6154 | -0.032 | 0.1072 | No |
| 77 | TGIF1 |  |  | 6360 | -0.036 | 0.0881 | No |
| 78 | CENPF |  |  | 6902 | -0.047 | 0.0353 | No |
| 79 | DDAH1 |  |  | 6943 | -0.049 | 0.0338 | No |
| 80 | TOP2A |  |  | 7678 | -0.069 | -0.0376 | No |
| 81 | ASPM |  |  | 7821 | -0.074 | -0.0483 | No |
| 82 | HIST1H4C |  |  | 8820 | -0.126 | -0.1436 | No |
| 83 | ARL6IP1 |  |  | 9038 | -0.146 | -0.1582 | No |
| 84 | MSMO1 |  |  | 9391 | -0.199 | -0.1837 | No |
| 85 | IRS2 |  |  | 9641 | -0.277 | -0.1947 | No |
| 86 | SOX2 |  |  | 9680 | -0.298 | -0.1829 | No |
| 87 | SOX4 |  |  | 9704 | -0.318 | -0.1686 | No |
| 88 | HMGCS1 |  |  | 9718 | -0.329 | -0.1527 | No |
| 89 | ID3 |  |  | 9778 | -0.389 | -0.1384 | No |
| 90 | SQLE |  |  | 9790 | -0.408 | -0.1182 | No |
| 91 | SAT1 |  |  | 9807 | -0.445 | -0.0965 | No |
| 92 | S100B |  |  | 9822 | -0.507 | -0.0714 | No |
| 93 | SCRG1 |  |  | 9879 | -1.473 | 0.0000 | No |
Table: GSEA details [plain text format]

  

Fig 2: FELDMAN\_2020\_LATEG1: Random ES distribution      
 Gene set null distribution of ES for **FELDMAN\_2020\_LATEG1**
